# Supplementary material for: Sustainable reference points for multispecies coral reef fisheries
Source: Nat Commun. 2023 Sep 4;14:5368. doi: 10.1038/s41467-023-41040-z (PMC10477311; doi:10.1038/s41467-023-41040-z)
Supplement: Supplementary file 3 — Description of Additional Supplementary Files [file 41467_2023_41040_MOESM3_ESM.docx]

**Description of Additional Supplementary Files**

File Name: Supplementary Data 1

Description: Reef site-scale data used in the main analyses.

File Name: Supplementary Data 2

Description: Jurisdiction-scale data used in the main analyses.

File Name: Supplementary Data 3

Description: Individual fish-scale data used to estimate total species richness and perform some supplementary analyses
