# Supplementary material for: Sustainable reference points for multispecies coral reef fisheries
Source: Nat Commun. 2023 Sep 4;14:5368. doi: 10.1038/s41467-023-41040-z (PMC10477311; doi:10.1038/s41467-023-41040-z)
Supplement: Supplementary file 7 — Reporting Summary [file 41467_2023_41040_MOESM7_ESM.pdf]

nature portfolio

Corresponding author(s): Jessica Zamborain Mason

Last updated by author(s): Jul 27, 2023

Reporting Summary

Nature Portfolio wishes to improve the reproducibility of the work that we publish. This form provides structure for consistency and transparency in reporting. For further information on Nature Portfolio policies, see our [Editorial Policies](#) and the [Editorial Policy Checklist](#).

Statistics

For all statistical analyses, confirm that the following items are present in the figure legend, table legend, main text, or Methods section.

n/a	Confirmed	
☐	☒	The exact sample size (*n*) for each experimental group/condition, given as a discrete number and unit of measurement
☐	☒	A statement on whether measurements were taken from distinct samples or whether the same sample was measured repeatedly
☐	☒	The statistical test(s) used AND whether they are one- or two-sided<br>*Only common tests should be described solely by name; describe more complex techniques in the Methods section.*
☐	☒	A description of all covariates tested
☐	☒	A description of any assumptions or corrections, such as tests of normality and adjustment for multiple comparisons
☐	☒	A full description of the statistical parameters including central tendency (e.g. means) or other basic estimates (e.g. regression coefficient) AND variation (e.g. standard deviation) or associated estimates of uncertainty (e.g. confidence intervals)
☒	☐	For null hypothesis testing, the test statistic (e.g. *F*, *t*, *r*) with confidence intervals, effect sizes, degrees of freedom and *P* value noted<br>*Give P values as exact values whenever suitable.*
☐	☒	For Bayesian analysis, information on the choice of priors and Markov chain Monte Carlo settings
☐	☒	For hierarchical and complex designs, identification of the appropriate level for tests and full reporting of outcomes
☐	☒	Estimates of effect sizes (e.g. Cohen's *d*, Pearson's *r*), indicating how they were calculated

Our web collection on [statistics for biologists](#) contains articles on many of the points above.

Software and code

Policy information about [availability of computer code](#)

Data collection	NA
Data analysis	Code used for this paper is available from GitHub (https://github.com/ JZamborain-Mason/ ZamborainMasonetal2023_ReefSustainability; DOI: 10.5281/zenodo.8190420. For the main analyses we used the RSTAN package (version 2.19.3). To account for methodological effects in our ecosystem response variables we used the brms package (version 2.18.0). Additional packages were used to re-arrange the data and for plotting purposes. All analyses were implemented in R (R version 4.2.1 (2022-06-23)).

For manuscripts utilizing custom algorithms or software that are central to the research but not yet described in published literature, software must be made available to editors and reviewers. We strongly encourage code deposition in a community repository (e.g. GitHub). See the Nature Portfolio [guidelines for submitting code & software](#) for further information.

Data

Policy information about [availability of data](#)

All manuscripts must include a [data availability statement](#). This statement should provide the following information, where applicable:

- Accession codes, unique identifiers, or web links for publicly available datasets
- A description of any restrictions on data availability
- For clinical datasets or third party data, please ensure that the statement adheres to our [policy](#)

For the main analyses of this study we compiled several existing datasets. We compiled three datasets on underwater reef associated fish and associated data (Cinner et al. 2020; MacNeil et al. 2015; McClanahan and Graham 2015). These used published species-specific length-weight relationships available from FishBase

(http://fishbase.org) to calculate reef fish biomass. Reconstructed reef fish catch estimates (in metric tonnes) were obtained from the Sea Around Us Project (SAUP) catch database (http:// www.seaaroundus.org). We also used the tropical coral reef spatial grid (https://data.unep-wcmc.org/datasets/1) to intersect with catch data and obtain site-specific and jurisdiction level reef fish catches. Additionally, several site-specific covariates in our model were obtained from on ine spatial data sources: human impact (https:/ /research.jcu.edu.au/data/published/a9167f52dba39f693f55ae68a0a5dccf/), sea surface temperature (https:// coralreefwatch.noaa.gov/) and ocean productivity (http://orca.science.oregonstate.edu/). Data used for this paper is available as Supplementary Data.

Human research participants

Policy information about studies involving human research participants and Sex and Gender in Research.

Reporting on sex and gender	NA
Population characteristics	NA
Recruitment	NA
Ethics oversight	NA

Note that full information on the approval of the study protocol must also be provided in the manuscript.

Field-specific reporting

Please select the one below that is the best fit for your research. If you are not sure, read the appropriate sections before making your selection.

☐ Life sciences ☐ Behavioural & social sciences ☒ Ecological, evolutionary & environmental sciences

For a reference copy of the document with all sections, see nature.com/documents/nr-reporting-summary-flat.pdf

Ecological, evolutionary & environmental sciences study design

All studies must disclose on these points even when the disclosure is negative.

Study description

This study aims to perform a global assessment of the sustainability status of the world's coral reef fisheries. First ,we estimate context-specific Multispecies Maximum Sustainable Yield (MMSY) reference points for coral reef assemblages using different aggregate surplus production models (e.g., Gompertz-Fox, Schaefer, Pella-Tomlinson) in a Bayesian framework. To do this, we used the trajectory of biomass in high compliance marine reserves (n=70) , the standing stock biomass in remote locations (n=80) and the biomass in fished reefs (n=1903) from catch-independent scientific stock assessments, as well as their environmental and methodological context (e.g., coral cover, sea surface temperature, ocean productivity, human impact, habitat) in a hierarchical structure. Secondly, we assessed the status of exploited coral reef fish stocks at a site (n=1903) and jurisdiction scale (n=111). This was done by using three sources of data: (i) standing stock biomass data from scientific stock assessments (n=1903), (ii) spatially reconstructed reef fish catch data from the Sea Around Us Project, and (iii) the grid of tropical coral reefs. We restricted the number of families and timing for both datasets to make them comparable. Finally, we characterized the potential trade-offs between production (in terms of the surplus production curve) and key metrics for coral reef ecosystems (mean fish length (n=1763), total fish species richness (n=1753), probability of encountering top predators(n=1763), and parrotfish scraping potential (n=1116)) using a hierarchical structure. This allowed us to assess the ecological consequences of having overfished stocks and the potential production and ecosystem benefits of increasing sustainability.

Research sample

Organism taxa: diurnally-active, non-cryptic reef fish above 10 cm length from families that are resident on the reef (Acanthuridae, Balistidae, Caesionidae, Carangidae, Chaetodontidae, Cirrhitidae, Diodontidae, Ephippidae, Haemulidae, Kyphosidae, Lethrinidae, Lutjanidae, Monacanthidae, Mullidae, Nemipteridae, Pinguipedidae, Pomacanthidae, Labridae, Serranidae, Siganidae, Sparidae, Sphyraenidae,cSynodontidae,Tetraodontidae and Zanclidae). The sample is meant to serve tropical multispecies reef fish populations available for harvest.
A total of 2053 coral reef sites were used (reefs had an average of 2.4 surveys (transects)) from 49 jurisdictions. Catch per unit area was extracted for sampled reef sites and a total of 108 jurisdictions.

Sampling strategy

No statistical methods were used to determine sample size. Sample was determined by data availability.

Data collection

No original data was collected as part of the study. However, several main global datasets were compiled:

(i) Standing stock biomass from scientific stock assessments: Individual fish were recorded through underwater visual census (UVC) from surveys spanning depths from 0 to 26 m and in all reef habitat types (i.e., slopes, crest, back reef and lagoons). All surveys used standard belt-transects, distance sampling, or point-counts. Methodology was accounted for in our modelling framework (see Randomization below). Within each survey area, diurnally-active, non-cryptic reef fish above 10 cm of length from families that are resident on the reef were retained, identified to species level, abundance counted, and total length (TL) estimated, except for one data provider who measured biomass at the family level. Biomass was calculated using published species-specific length–weight relationships available on FishBase (http://fishbase.org). When length–weight relationship parameters were not available for a species, we used the parameters for a closely related species, or larger taxonomic level (e.g., genus or family). We extracted socio-ecological data for those sites from other sources(see Data section above).

	(ii) Spatially reconstructed reef fish catch per unit area: Reconstructed reef fish catch estimates (in metric tonnes) were obtained from the Sea Around Us Project (SAUP) catch database (http://www.seaaroundus.org). We only used fish classified as "reef associated" species of the families included in our biomass estimates. We calculated the catch per unit area by intersecting the catch and the reef area (UNDP-WCMC) polygons. A jurisdiction's catch per unit area was estimated by summing the total reef associated catch for that jurisdiction and dividing it by the estimated reef area.
Timing and spatial scale	Standing stock information covered 2053 reefs from 49 tropical jurisdictions around the tropical globe, giving estimates of reef fish at the scale of kg/ha.<br>To estimate the sustainable reference points we required the biomass trajectory in marine reserves (i.e., data covering a range of marine reserve ages). For this purpose, we used standing stock biomass surveys conducted between 1999 and 2014.<br>For the rest of the manuscript (i.e., assessment and ecosystem metrics) we included only standing stock data from the year closest to 2010. This covered surveys conducted between 2004 and 2014.<br><br>Catch per unit area was available at a jurisdiction level for 111 tropical jurisdictions around the global tropics. Note only 108 had spatial reconstructed catch data per unit area. The remaining (only in supporting information) had jurisdiction level catch per unit area but not spatial. This data was collected for the period between 2008 and 2014.
Data exclusions	Reefs in our standing stock biomass data were classified into different management groups: (i) openly fished (i.e., regularly fished without effective restrictions), (ii) restricted fishing - whether there were active restrictions on gears (e.g., bans on the use of nets, spearguns, or traps) or fishing effort (e.g., bag limits), (iii) high compliance no-take marine reserves, and (iv) low compliance marine reserves (e.g., paper parks). For the purpose of our study, we excluded low compliance marine reserves from the analyses.
Reproducibility	All attempts to reproduce the analyses were successfull (running analyses each time we received reviews)
Randomization	Sites were assigned to management groups based on data provider classifications and a metric of travel time. Except for the biomass trajectory of reserve reefs, where data from multiple years were available from a single reef, we included only data from the year closest to 2010. If a reserve was sampled multiple times (i.e., at different ages), we randomly chose one year and checked that the randomly selected years did not affect the robustness of our MMSY parameter estimates. Additionally, standing stock response variables were corrected for methodological covariates (Habitat type, Depth, Census method, and Sampling area) using a spatially hierarchical model and calculating the marginalized biomass using slopes, standard belt transects, 4-10 m depth and average sampling area as a reference.
Blinding	Blinding was not relevant for this study (e.g., a posteriori observational study)

Did the study involve field work? ☐ Yes ☒ No

Reporting for specific materials, systems and methods

We require information from authors about some types of materials, experimental systems and methods used in many studies. Here, indicate whether each material, system or method listed is relevant to your study. If you are not sure if a list item applies to your research, read the appropriate section before selecting a response.

Materials & experimental systems

n/a	Involved in the study
☒	☐ Antibodies
☒	☐ Eukaryotic cell lines
☒	☐ Palaeontology and archaeology
☒	☐ Animals and other organisms
☒	☐ Clinical data
☒	☐ Dual use research of concern

Methods

n/a	Involved in the study
☒	☐ ChIP-seq
☒	☐ Flow cytometry
☒	☐ MRI-based neuroimaging
